# Tentacle extract from the jellyfish *Cyanea capillata* increases proliferation and migration of human umbilical vein endothelial cells through the ERK1/2 signaling pathway

**DOI:** 10.1371/journal.pone.0189920

**Published:** 2017-12-20

**Authors:** Beilei Wang, Dan Liu, Chao Wang, Qianqian Wang, Hui Zhang, Guoyan Liu, Qian He, Liming Zhang

**Affiliations:** 1 Marine Bio-pharmaceutical Institute, Second Military Medical University, Shanghai, China; 2 Department of Marine Biotechnology, Faculty of Naval Medicine, Second Military Medical University, Shanghai, China; 3 Department of Urology, Shanghai Changhai Hospital, Second Military Medical University, Shanghai, China; 4 Department of Gynecology, Third Affiliated Hospital, Second Military Medical University, Shanghai, China; Duke University School of Medicine, UNITED STATES

## Abstract

Wound healing is a complex biological process, and current research finds that jellyfish have a great capacity for promoting growth and healing. However, the underlying mechanisms remain unclear. Thus, this study was conducted to investigate the molecular mechanisms and effects of a tentacle extract (TE) from the jellyfish *Cyanea capillata* (*C*. *capillata*) on cell proliferation and migration in human umbilical vein endothelial cells (HUVECs). First, our results showed that TE at the concentration of 1 μg/ml could promote cell proliferation over various durations, induce a transition of the cells from the G1-phase to the S/G2-phase of the cell cycle, and increase the expression of cell cycle proteins (CyclinB1 and CyclinD1). Second, we found that TE could activate the PI3K/Akt, ERK1/2 and JNK MAPK signaling pathways but not the NF-κB signaling pathway or the apoptosis signaling cascade. Finally, we demonstrated that the TE-induced expression of cell cycle proteins was decreased by ERK1/2 inhibitor PD98059 but not by PI3K inhibitor LY294002 or JNK inhibitor SP600125. Similarly, the TE-enhanced migration ability of HUVECs was also markedly attenuated by PD98059. Taken together, our findings indicate that TE-induced proliferation and migration in HUVECs mainly occurred through the ERK1/2 MAPK signaling pathway. These results are instructively important for further research on the isolation and purification of growth-promoting factors from *C*. *capillata* and are hopeful as a means to improve human wound repair in unfavorable conditions.

## 1. Introduction

In the ocean, marine organisms suffer from various injuries from relatively minor, such as flesh wounds caused by unsuccessful attacks, to severe events, such as losing limbs or shark finning. The proportion of injured individuals in marine benthic invertebrate populations has been reported to range from 33% to 47% at a given time [1]. However, many marine organisms, especially marine benthic invertebrates, have evolved some unique self-repair or regeneration abilities in response to these life-threating stressors. The marine organisms that possess a strong regenerative capacity include jellyfish, sponges, corals, sea anemones, starfish, ctenophores, clams, polychaetes, and brittlestars [1–3]. Among them, jellyfish (Scyphozoa), which are a class of cnidarians, have gained increasing attention for their complex life cycles and as an abundant resource [4–6]. According to some reports, although jellyfish are often attacked by some predators, such as sea turtles, parts of the jellyfish body, especially the threadlike tentacles, are easily broken off, and the damaged area will grow new tentacles without any visible scar tissue [2, 7]. In addition, jellyfish also show a quick response to environmental changes by enhancing their feeding, growth and reproduction [6]. For example, many jellyfish exhibit a maximum growth rate of ~3 mm d^-1^ (± 0.2 mm d^-1^) [8] and instantaneous growth rates exceed 0.3 d^-1^ [9], which are possibly the highest ever recorded in epi-pelagic metazoans. Moreover, cnidarians are reported to have the potential to undergo regenerative, asexual-reproduction and even reverse development [4, 10]. The switch is achieved by a variable combination of cellular processes such as cell proliferation and transdifferentiation [10]. In recent years, we have been committed to exploring the bioactive substances from the jellyfish *Cyanea capillata* (*C*. *capillata*), which is also known as the lion’s mane jellyfish or the hair jellyfish [11, 12]. This jellyfish is one of the most common venomous jellyfish in the East China Sea, and jellyfish populations have been increasing over recent years. To obtain an overview of the bioactive components of *C*. *capillata*, we previously performed a transcriptome analysis and identified some transcripts, such as vascular endothelial growth factors (VEGFs), in the jellyfish tentacles. Thus, we believe that growth-promoting components exist in the tentacles of *C*. *capillata* and that a constant exploration of these novel compounds would possibly promote the development of marine-derived drugs for wound repair in humans.

It is known that wound healing is a complex biological process that involves growth factors, cytokines and other mediators [3, 13, 14]. After an injury, multiple biological events of cell proliferation, migration and extracellular matrix deposition are activated in response to tissue damage [15]. Most of the wounds naturally heal, but some problematic conditions, including diabetes [16], ischemia [17], infection [16] and radiation exposure [18], can contribute to healing failure. Hence, it is imperative to develop novel bioactive molecules or beneficial engineered tissue alternatives to treat these non-healing wound conditions [19]. The current research in this field has fully proven that marine-derived biological macromolecules such as collagen, alginate, chitin, chitosan, fucoidan and carrageenan have significant abilities to enhance the healing process and reestablish skin tissue [19, 20]. However, until now, the mechanism underlying the growth- and healing-promoting capacities in jellyfish has remained unclear. Therefore, in the present study and using tentacle extract (TE) from *C*. *capillata* as the sample, we aimed to evaluate the effects of TE on the proliferation and migration in human umbilical vein endothelial cells (HUVECs). Furthermore, we attempted to elucidate the possible mechanism of the bioactivity of TE by focusing on the PI3K/Akt, MARK and NF-κB signaling pathways.

## 2. Materials and methods

### 2.1. Reagents

A Cell Counting Kit-8 (CCK-8) was purchased from Dojindo Molecular Technologies (Kumamoto, Japan). The antibodies against phospho-Akt (Ser473), Akt, phospho-JNK (Thr183/Tyr185), JNK, phospho-p38, p38, phospho-NF-κB (Ser536), NF-κB, phospho-IκBα and IκBα were purchased from Cell Signaling Technology (Beverly, MA, USA). Phospho-ERK1/2, ERK1/2, caspase-3, caspase-8, caspase-9, CytoC and GAPDH antibodies were purchased from Abcam (Cambridge, MA, USA). Beta-Tubulin, HRP-conjugated anti-rabbit IgG and anti-mouse IgG were purchased from Beyotime (Jiangsu, China). LY294002 (PI3K/Akt inhibitor), PD98059 (ERK1/2 inhibitor), SP600125 (JNK inhibitor) and Bay11-7082 (NF-κB inhibitor) were purchased from Selleck (Houston, USA). FITC-conjugated goat anti-rabbit IgG and FITC-conjugated goat anti-mouse IgG were purchased from EarthOx Life Sciences (Millbrae, CA, USA). The 4’,6-diamidino-2-phenylindole (DAPI) was purchased from Beyotime (Jiangsu, China).

### 2.2. TE preparation from the jellyfish *C*. *capillata*

Specimens of *C*. *capillata* collected in July 2015 in the Sanmen Bay, East China Sea, were identified by Professor Huixin Hong from the Fisheries College of Jimei University, Xiamen, China. Catching of the jellyfish was permitted by the East China Sea Branch, State Oceanic Administration, People’s Republic of China. The removed tentacles were placed in plastic bags on dry ice and immediately shipped to Shanghai, where the samples were frozen at -80°C until ready for use. The TE was prepared following the method as described in our previous reports [11, 12]. Briefly, the frozen tentacles of *C*. *capillata* were thawed at 4°C and immersed in filtered seawater at a mass/volume ratio of 1:1 to allow autolysis of the tissues for 4 days. The mixture was stirred for 30 min twice daily. The autolyzed mixture was centrifuged at 10,000 × *g* for 15 min thrice. The resultant supernatant was the TE. All the procedures were performed at 4°C or in an ice bath. The TE was centrifuged at 10,000 × *g* for 15 min to remove the sediments and followed by dialysis against phosphate buffered saline (PBS, 0.01 mol/L, pH 7.4) for over 8 h before use. The protein concentration in the preparations was determined using the method of Bradford.

### 2.3. Cell culture

The human umbilical vein endothelial cells (HUVECs) cell line was purchased from Zhongqiaoxinzhou Biotech (Shanghai, China) and grown in high-glucose DMEM medium (HyClone, Waltham, MA, USA) supplemented with 10% fetal calf serum (FBS, Gibco, USA), 100 U/ml penicillin, and 100 μg/ml streptomycin at 37°C in a humidified incubator with 95% air and 5% CO_2_.

### 2.4. Cell viability assay

The cell viability was determined by the CCK-8 assay. HUVECs were plated in 96-well culture plates at a density of 1–2 × 10^4^ cells/ml. After incubation for 24 h, the cells were treated with TE (0–24 μg/ml) for 1 h and 6 h. Then, 10 μl of CCK-8 reagents were added to each well for 4 h at 37°C. The absorbance at 450 nm was measured using a microplate reader (BioTek, Winooski, VT, USA). The percentage of the cell growth was calculated using the following formula:

$$\text{Proliferation}\;\left(\%\right)=\left(1-{\text{OD}}_{\text{treated}}/{\text{OD}}_{\text{untreated}}\right)\times 100.$$

### 2.5. Cell cycle analysis

HUVECs, which were seeded in 6-well culture plates (3 × 10^5^ cells/well), were treated with TE (1 μg/ml) for different durations (0–360 min). Then, the trypsin-harvested cells were fixed in ice-cold 75% ethanol at 4°C for 30 min. The cells were washed twice with PBS at room temperature and then incubated with the staining solution containing PI (50 μg/ml) and Rnase A (50 μg/ml) for 30 min. The fluorescence was measured and analyzed using an FAC Scan flow cytometer (Becton Dickinson, San Jose, CA, USA).

### 2.6. Western blotting

To investigate the time-effect relationship of TE on the cell viability, HUVECs were treated with TE (1 μg/ml) for different durations (0–60 min). To explore the effect of TE on phosphorylation of signaling proteins, HUVECs were treated with TE (1 μg/ml) for different durations (0–60 min) in the presence or absence of LY294002 (10 μM), PD98059 (10 μM), SP600125 (10 μM) and Bay11-7082 (10 μM). To survey the effect of TE on the expression of cell cycle proteins, HUVECs were pretreated with TE (1 μg/ml) for different time durations (0–360 min) in the presence or absence of LY294002 (10 μM), PD98059 (10 μM) and SP600125 (10 μM).

After treatments, the cells were lysed on ice in RIPA buffer with a protease inhibitor (1% PMSF). The protein content was measured by the Bradford assay. Equal amounts of protein per sample were loaded on a 12% SDS-PAGE gel and then transferred to a polyvinylidene difluoride or nitrocellulose membrane, which was then blocked with 5% non-fat dry milk in TBST (3 g of Tris-base, 8 g of NaCl, 0.2 g of KCl, 0.05% Tween-20, diluted with water to 1000 ml, pH 7.4) for 2 h at room temperature. The membranes were then incubated overnight at 4°C with primary antibodies as follows: p-Akt (1:1000), Akt (1:1000), p-ERK1/2 (1:1000), ERK1/2 (1:1000), p-JNK (1:500), JNK (1:500), phospho-p38 (1:500), p38 (1:500), p-NF-κB (1:1000), NF-κB (1:1000), p-IκBα (1:500), caspase-3 (1:250), caspase-8 (1:250), caspase-9 (1:250), CytoC (1:500), CyclinB1 (1:1000), CyclinD1 (1:1000), GAPDH (1:5000) and beta-Tubulin (1:1000), with gentle shaking. The ECL method was used with secondary antibodies (HRP-conjugated anti-rabbit IgG and anti-mouse IgG) at a dilution of 1:5,000 for 2 h at room temperature. Then, the membranes were exposed using a chemiluminescent detection system (Syngene G: Box, USA). Quantitative densitometric analyses of immunoblots were performed using ImageJ software (Ver. 1.48, Bethesda, MD, USA), and the relative ratio was calculated.

### 2.7. Immunofluorescence staining

HUVECs were grown on coverslips located in 6-well culture plates. The control group was incubated with PBS, and other groups were incubated with TE (1 μg/ml) in the presence or absence of LY294002 (20 μM), PD98059 (20 μM) and SP600125 (20 μM). After the treatments, HUVECs were fixed with a 4% paraformaldehyde solution for 30 min and then permeabilized with a 0.5% Triton X-100 solution (in PBS) for 5 min at room temperature. Subsequently, the HUVECs were blocked with 5% BSA (in PBS) for 30 min at room temperature and incubated overnight at 4°C with primary antibodies as follows: p-Akt (1:100), p-ERK1/2 (1:100), p-JNK (1:100), CyclinB1 and CyclinD1 (1:200). After incubation with the primary antibodies, the HUVECs were further incubated with FITC-labeled goat anti-rabbit or anti-mouse antibody for 3 h and then incubated with DAPI (10 μg/ml) for 1 min at room temperature. Afterward, the coverslips were sealed onto the slides with antifade mountants. Finally, the fluorescence images were visualized with a confocal laser scanning microscope (Olympus FV 1000, Japan) with excitation and emission wavelengths of 492 nm and 520 nm, respectively. In addition, the fluorescence intensities were obtained from the dataset of the images using the FV10-ASW 3.1 software (Olympus FV 1000, Japan).

### 2.8. Cell migration assay (wound healing assay)

HUVECs were seeded in 6-well culture plates (3 × 10^5^ cells/well) and grown to confluence. After serum starvation, the cells were scraped to create a wound area in the center of the cell monolayers (the time point set as 0 h). Afterward, the cells were washed with PBS and then incubated with TE (1 μg/ml) in the presence or absence of PD98059 (20 μM) for 24 h. The wound closure was photographed at different durations (0, 12, and 24 h) at 100 ×. The extent of the wound closure was quantitated by calculating the difference in the denuded area using ImageJ software (Ver. 1.48, Bethesda, MD, USA).

### 2.9. Statistical analysis

In the experiments, the images shown are representative of at least three experiments performed on different experimental days. The data are presented as the means ± SEM. Analysis of variance (One-way ANOVA) and the Bonferroni post hoc test were used in the statistical evaluation of the data as appropriate. A value of *P* < 0.05 was considered significant.

## 3. Results

### 3.1. Effects of TE on the viability of HUVECs

**Fig 1:**
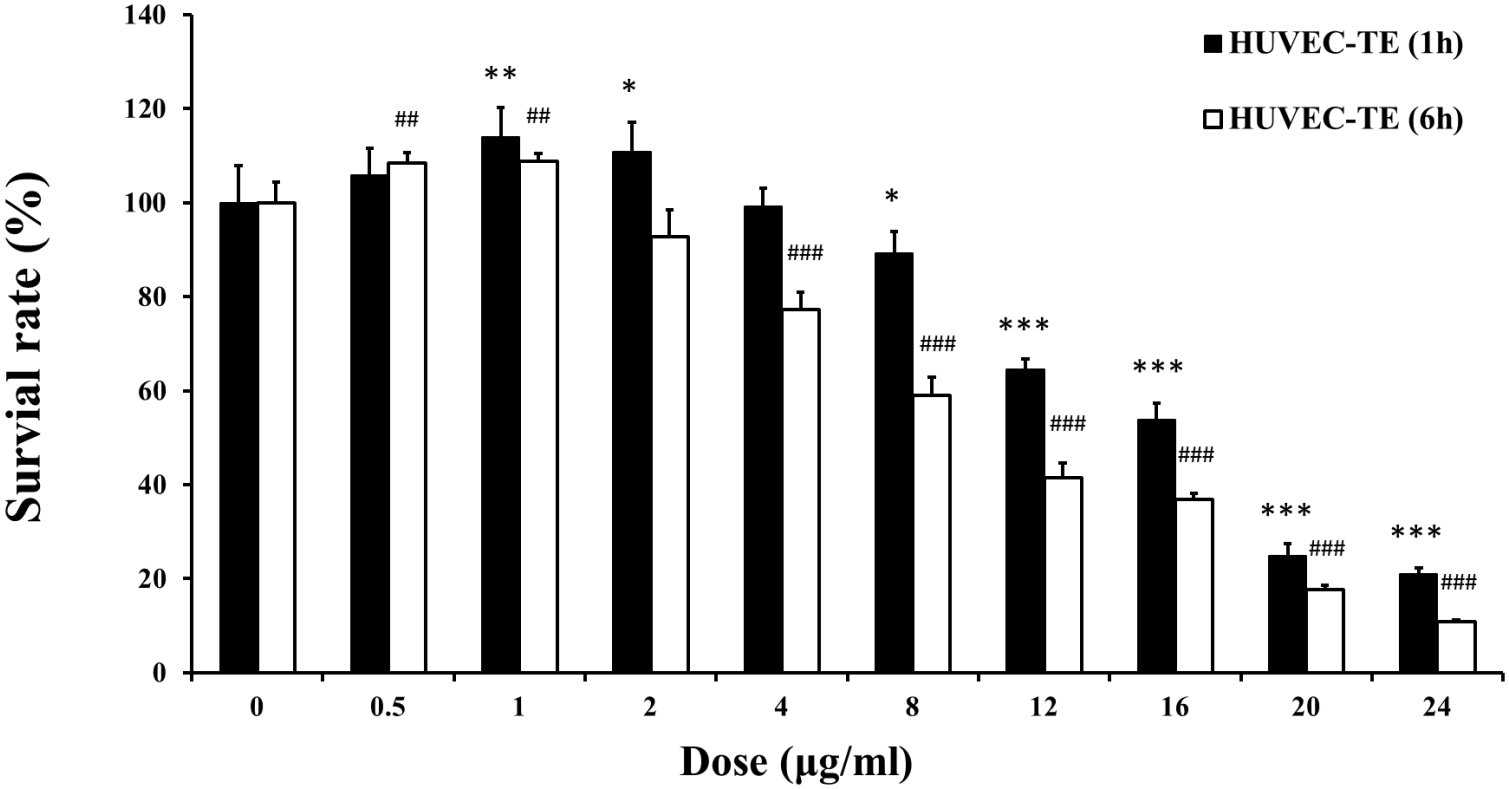
Effects of TE on the viability of HUVECs. HUVECs were stimulated with different doses of TE (0–24 μg/ml) for 1 h and 6 h. *P < 0.05, **P < 0.01, ***P < 0.001 *vs*. Control (1 h); ^##^*P* < 0.01, ^###^*P* < 0.001 *vs*. Control (6 h).

As shown in Fig 1, when HUVECs were treated with various doses of TE for 1 h, the cell viability decreased at concentrations of 8–24 μg/ml in a dose-dependent manner with an IC_50_ value of 19.26 μg/ml. However, TE at 4 μg/ml did not significantly influence the cell viability after 1 h of treatment. Interestingly, the cell viability increased at TE concentrations of 0.5–2 μg/ml, and the increase of cell viability was significant at TE concentrations of 1–2 μg/ml. Similarly, after treatment with TE for 6 h, the cell viability decreased at TE concentrations of 4–24 μg/ml in a dose-dependent manner with an IC_50_ value of 15.67 μg/ml. However, TE did not significantly influence the cell viability at 2 μg/ml, but it markedly increased the cell viability at concentrations of 0.5–1 μg/ml. Therefore, the TE dose of 1 μg/ml, which effectively promoted the proliferation of HUVECs, was selected for the next study.

### 3.2. Effects of TE on the cell cycle of HUVECs

#### 3.2.1. Effects of TE on the cycle progression of HUVECs

**Fig 2:**
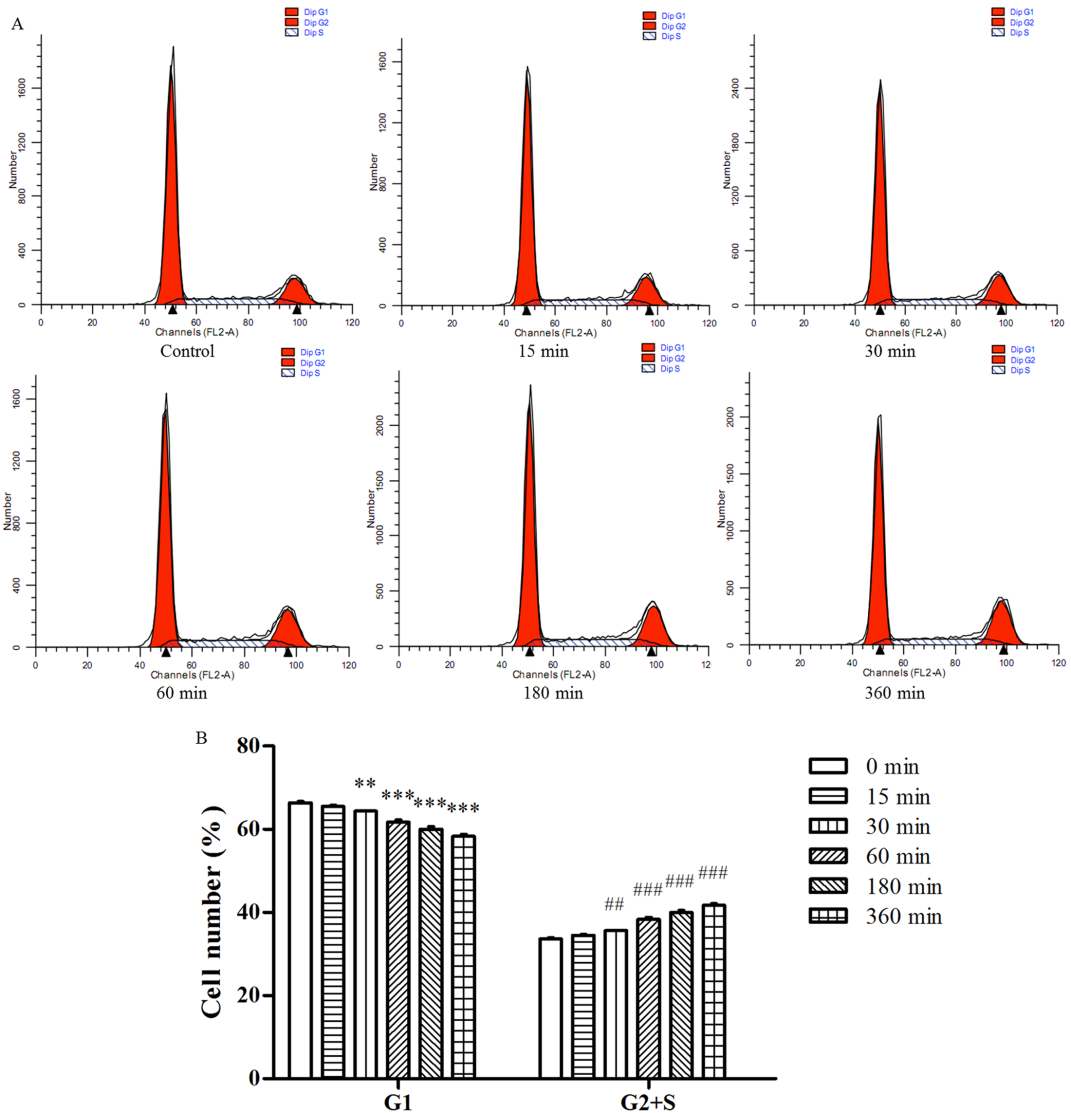
Effects of TE on the cell cycle progression of HUVECs. A: Representative histograms showing cell cycle distribution upon TE (1 μg/ml) for different times. B: Bar graph summarizing cell cycle progression data. **P < 0.01, ***P < 0.001 *vs*. Control of G1-phase; ^##^*P <* 0.01, ^###^*P <* 0.001 *vs*. Control of S/G2 –phase.

To further elucidate the mechanism of TE promoting the proliferation of HUVECs, the effects of TE on the cell cycle progression were examined by flow cytometry. As shown in Fig 2A, TE (1 μg/ml) induced the transition of cells from the G1-phase to S/G2-phase in a time-dependent manner. The cell cycle analysis showed a significant decrease in the cells from 66.38% to 58.33% in the G1-phase and an increase from 33.62% to 41.57% in S/G2-phase (Fig 2B). These results indicated that TE might promote the transition of HUVECs from the G1-phase to the S/G2-phase, which ultimately promoted cell proliferation.

#### 3.2.2. Effects of TE on the cell cycle protein expression of HUVECs

**Fig 3:**
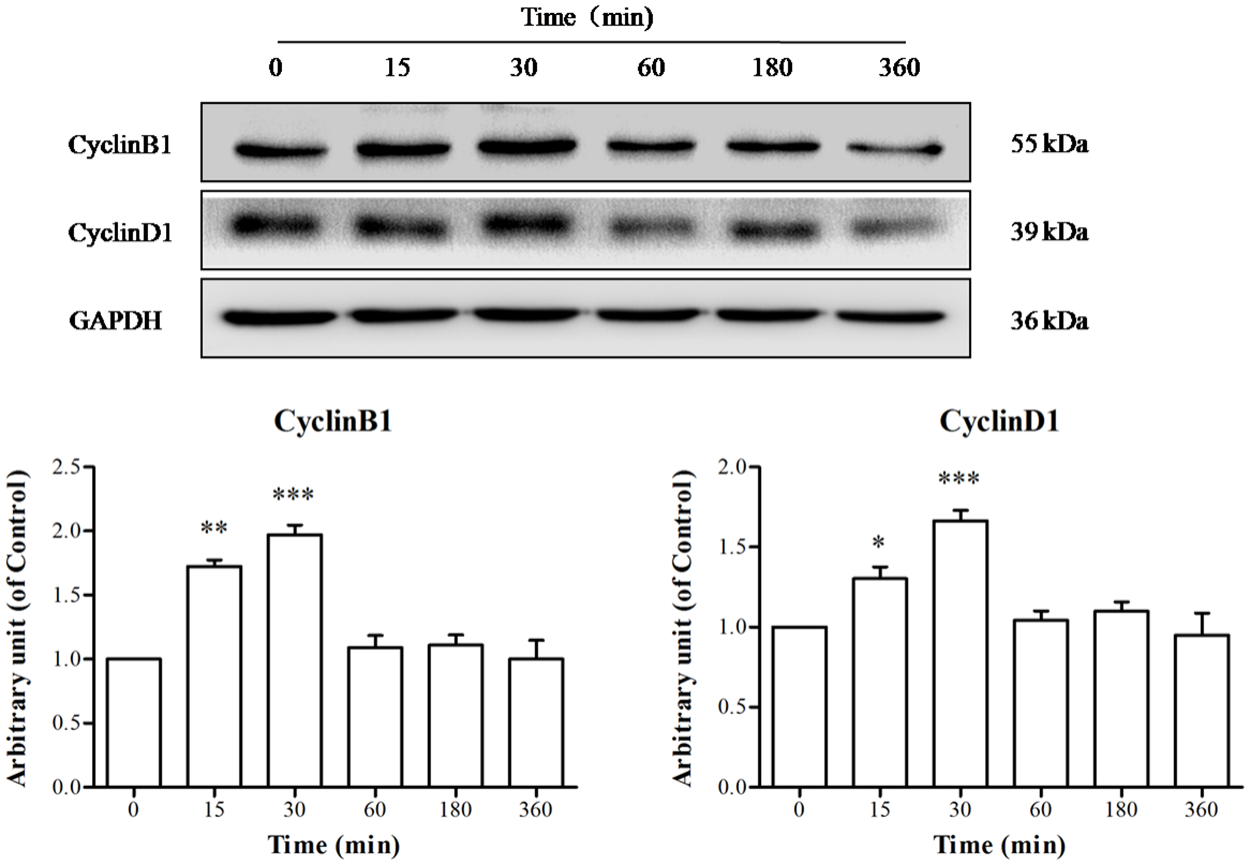
Effects of TE on the expression of cell cycle proteins (CyclinB1 and CyclinD1) in HUVECs. *P < 0.05, ***P < 0.001 *vs*. Control.

As shown in Fig 3, the expression of CyclinB1 increased significantly from 15 to 30 min after TE (1 μg/ml) treatment, with the maximum expression occurring at approximately 30 min. Similarly, the expression of CyclinD1 was also induced by TE (1 μg/ml), with a maximum at approximately 30 min. These results indicated that TE could activate the expression of cell cycle proteins.

### 3.3. Effects of TE on the cell signaling pathways in HUVECs

#### 3.3.1. Effects of TE on the cell signaling pathways in HUVECs by western blotting

**Fig 4:**
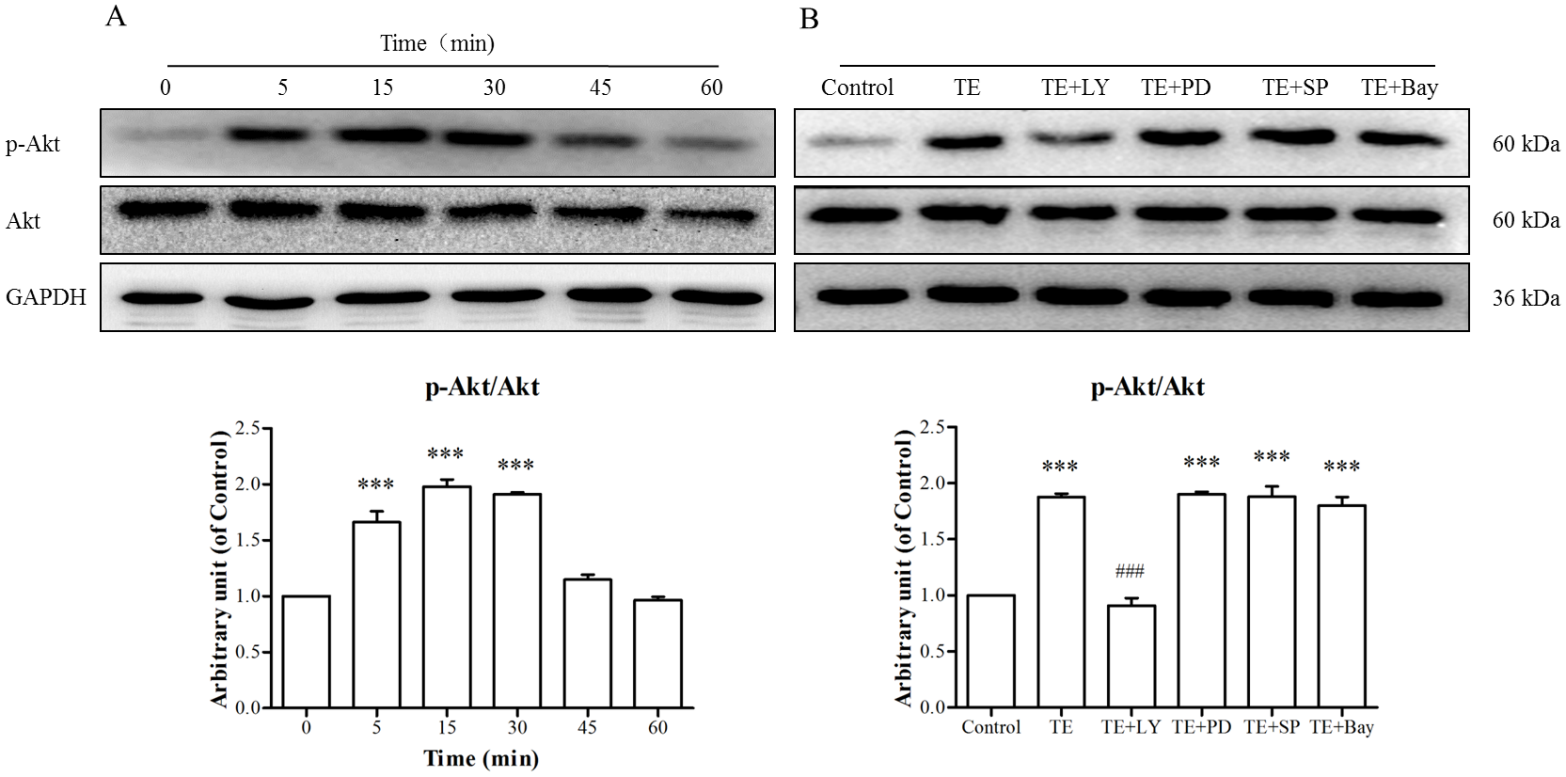
Responses of the PI3K/Akt signaling pathway to TE (1 μg/ml). A: HUVECs were treated with TE (1 μg/ml) for various durations. B: HUVECs were treated with TE (1 μg/ml) in the presence or absence of various signaling pathway inhibitors for 15 min. LY: LY294002, PD: PD98059, SP: SP600125, Bay: Bay11-7082; *P < 0.05, **P < 0.01, ***P < 0.001 *vs*. Control; ^###^*P <* 0.001 *vs*. TE.

As shown in Fig 4A, the phosphorylation of Akt was markedly induced by TE (1 μg/ml) from 5 to 45 min, with a maximum phosphorylation occurring at approximately 15 min, whereas TE seemed to have no effect on the level of total Akt. However, we pretreated HUVECs with PI3K, ERK1/2, JNK MARK and NF-κB signaling inhibitors before TE presence to confirm whether the PI3K/Akt signaling pathway was involved. Our results (Fig 4B) showed that the PI3K inhibitor LY294002 completely blocked TE-induced Akt phosphorylation, whereas other signaling pathway inhibitors, including PD98059, SP600125 and Bay11-7082, had no effect. These results indicate that TE could activate the PI3K/Akt signaling pathway.

**Fig 5:**
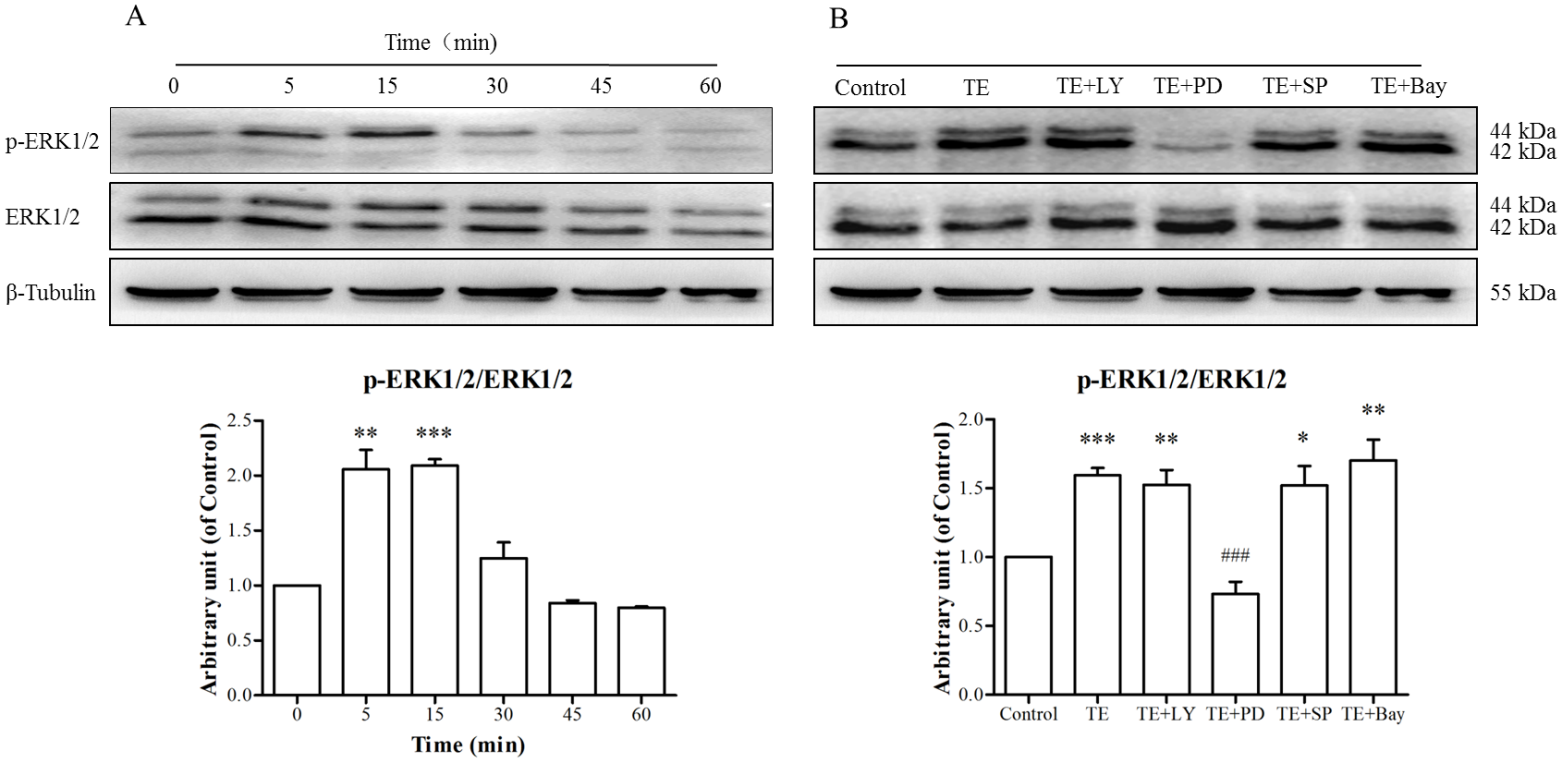
Responses of the ERK1/2 MAPK signaling pathway to TE (1 μg/ml). A: HUVECs were treated with TE (1 μg/ml) for various durations. B: HUVECs were treated with TE (1 μg/ml) in the presence or absence of various signaling pathway inhibitors for 15 min. LY: LY294002, PD: PD98059, SP: SP600125, Bay: Bay11-7082; *P < 0.05, **P < 0.01, ***P < 0.001 *vs*. Control; ^###^*P <* 0.001 *vs*. TE.

As shown in Fig 5A, the phosphorylation of ERK1/2 was markedly induced by TE (1 μg/ml) from 5 to 15 min, with the maximum phosphorylation occurring at approximately 15 min, whereas TE seemed to have no effect on the level of total ERK1/2. Then, we pretreated HUVECs with PI3K, ERK1/2, JNK MARK and NF-κB signaling inhibitors before TE presence to confirm whether the ERK1/2 MARK signaling pathway was involved. The results (Fig 5B) showed that the ERK1/2 MAPK inhibitor PD98059 completely blocked TE-induced ERK1/2 phosphorylation, whereas other signaling pathway inhibitors, including LY294002, SP600125 and Bay11-7082, had no effect. These results indicate that TE could activate the ERK1/2 MAPK signaling pathway.

**Fig 6:**
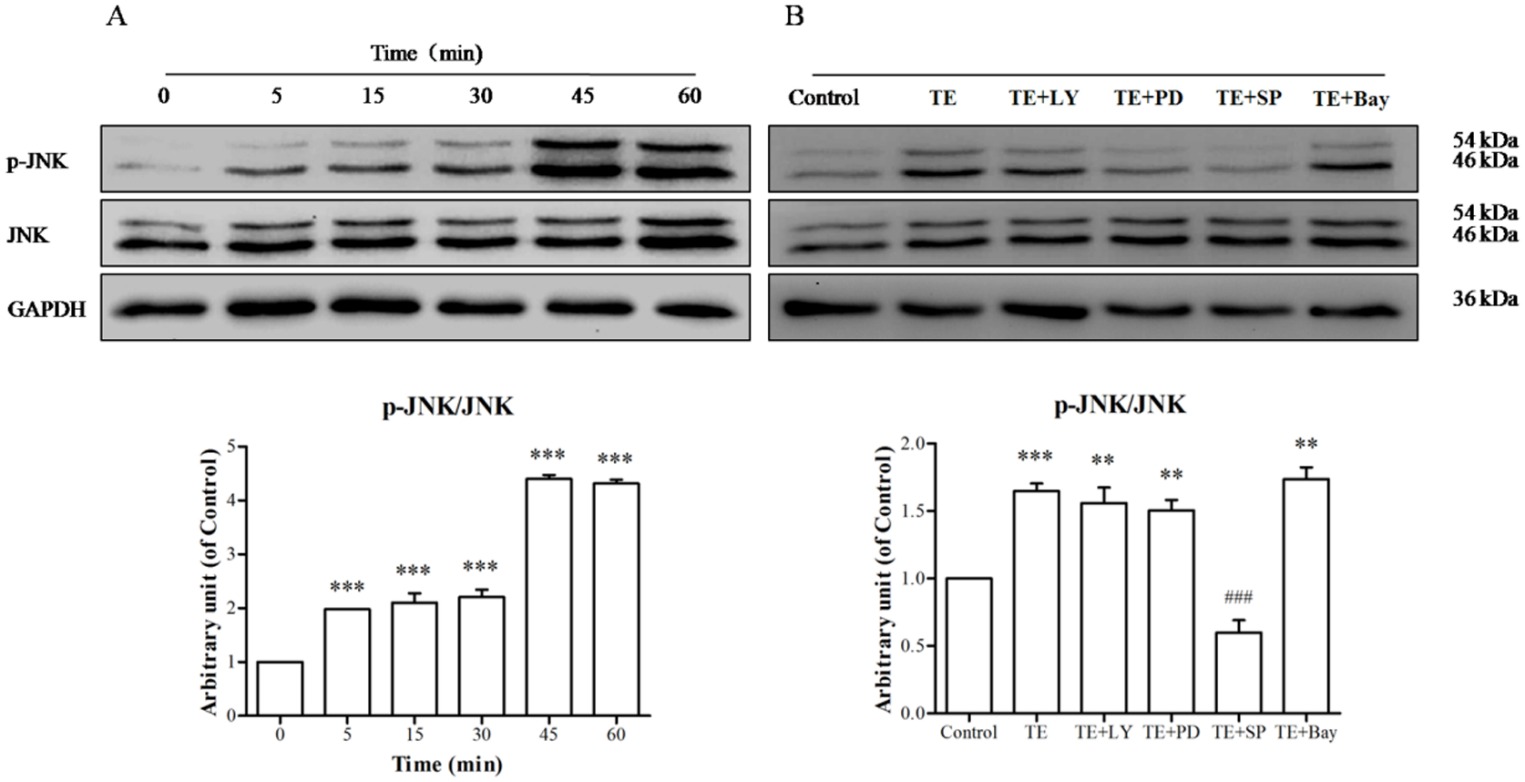
Responses of the JNK MAPK signaling pathway to TE (1 μg/ml). A: HUVECs were treated with TE (1 μg/ml) for various durations. B: HUVECs were treated with TE (1 μg/ml) in the presence or absence of signaling pathway inhibitors for 15 min. LY: LY294002, PD: PD98059, SP: SP600125, Bay: Bay11-7082; *P < 0.05, **P < 0.01, ***P < 0.001 *vs*. Control; ^###^*P <* 0.001 *vs*. TE.

As shown in Fig 6A, the phosphorylation of JNK was markedly induced by TE (1 μg/ml) from 5 to 60 min, with maximum phosphorylation occurring at approximately 45 min, whereas TE seemed to have no effect on the level of total JNK. However, we pretreated HUVECs with PI3K, ERK1/2, JNK MARK and NF-κB signaling inhibitors before TE presence to confirm whether the JNK MAPK signaling pathway was involved. Our results (Fig 6B) showed that the JNK MAPK inhibitor SP600125 completely blocked TE-induced JNK phosphorylation, whereas other signaling pathway inhibitors, including LY294002, PD98059 and Bay11-7082, had no effect. These results indicate that TE could activate the JNK MAPK signaling pathway.

**Fig 7:**
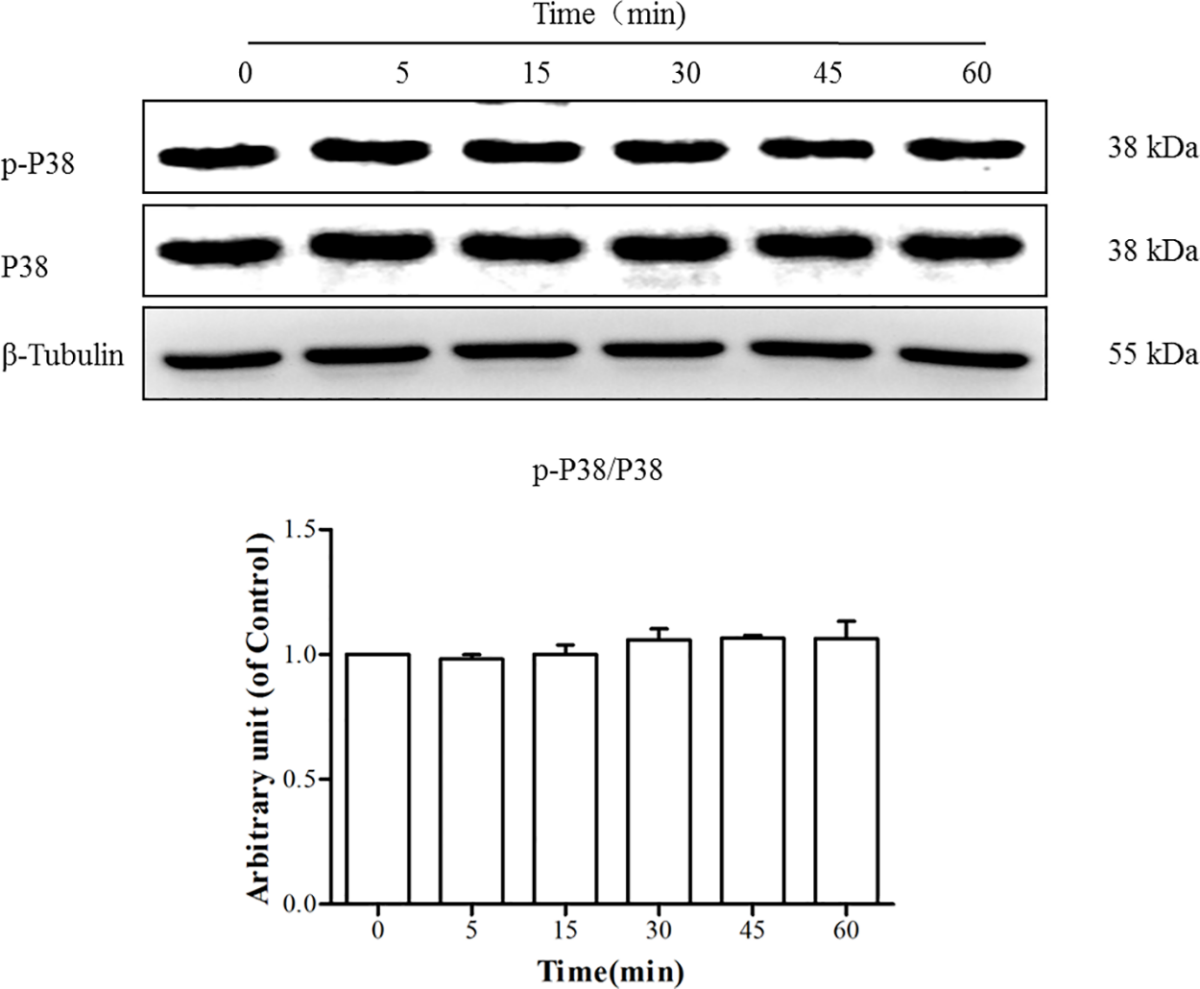
Responses of the p38 MAPK signaling pathway to TE (1 μg/ml). HUVECs were treated with TE (1 μg/ml) for various durations.

As shown in Fig 7, TE seemed to have no effect on the level of p-p38 or total p38, which indicated that TE could not activate the p38 MAPK signaling pathway.

**Fig 8:**
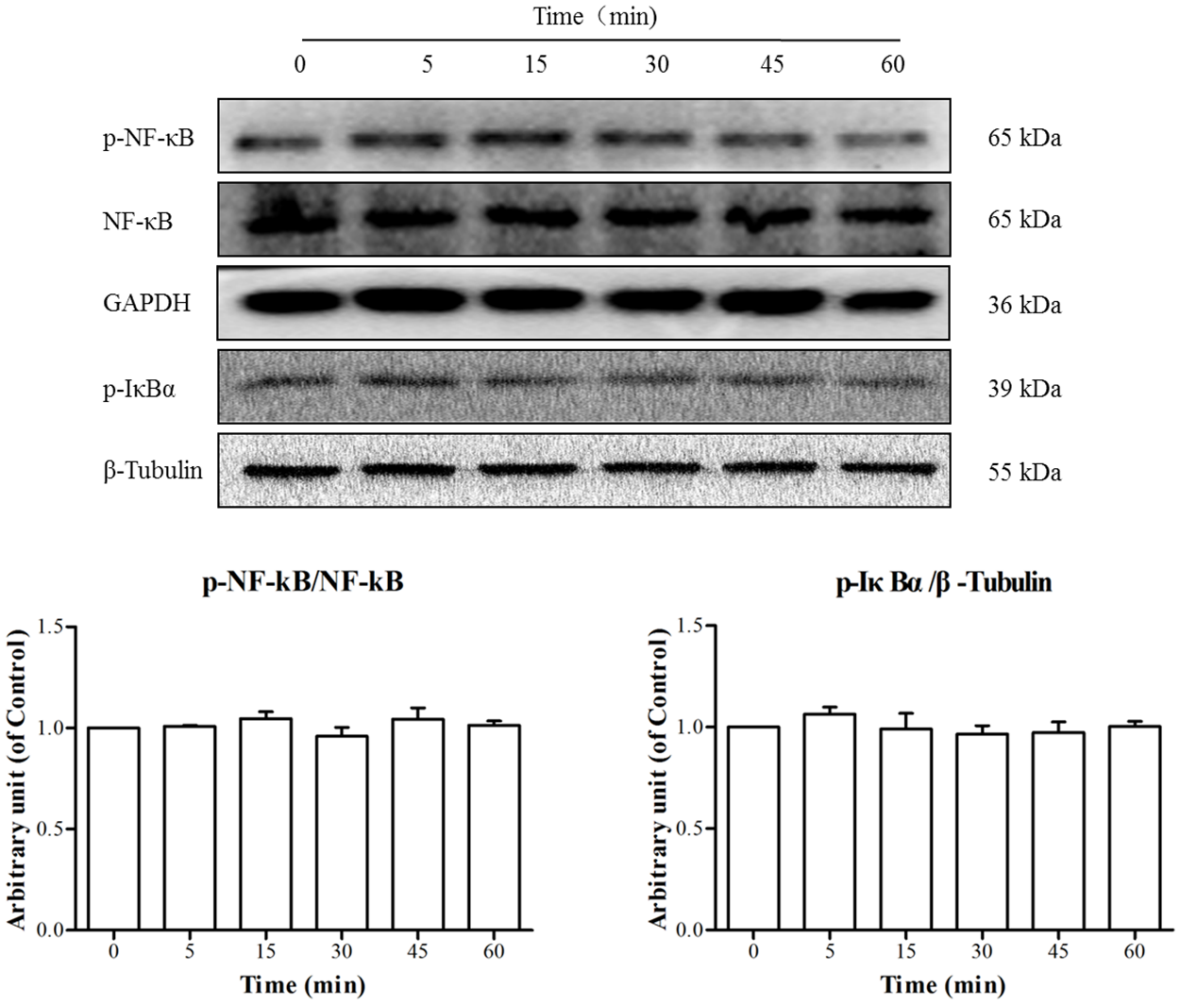
Responses of the NF-κB signaling pathway to TE (1 μg/ml). HUVECs were treated with TE (1 μg/ml) for various durations.

As shown in Fig 8, TE seemed to have no effect on the level of p-NF-κB or total NF-κB. Similarly, the expression of p-IκBα showed no significant change by TE either. These results indicate that TE could not activate the NF-κB signaling pathway.

**Fig 9:**
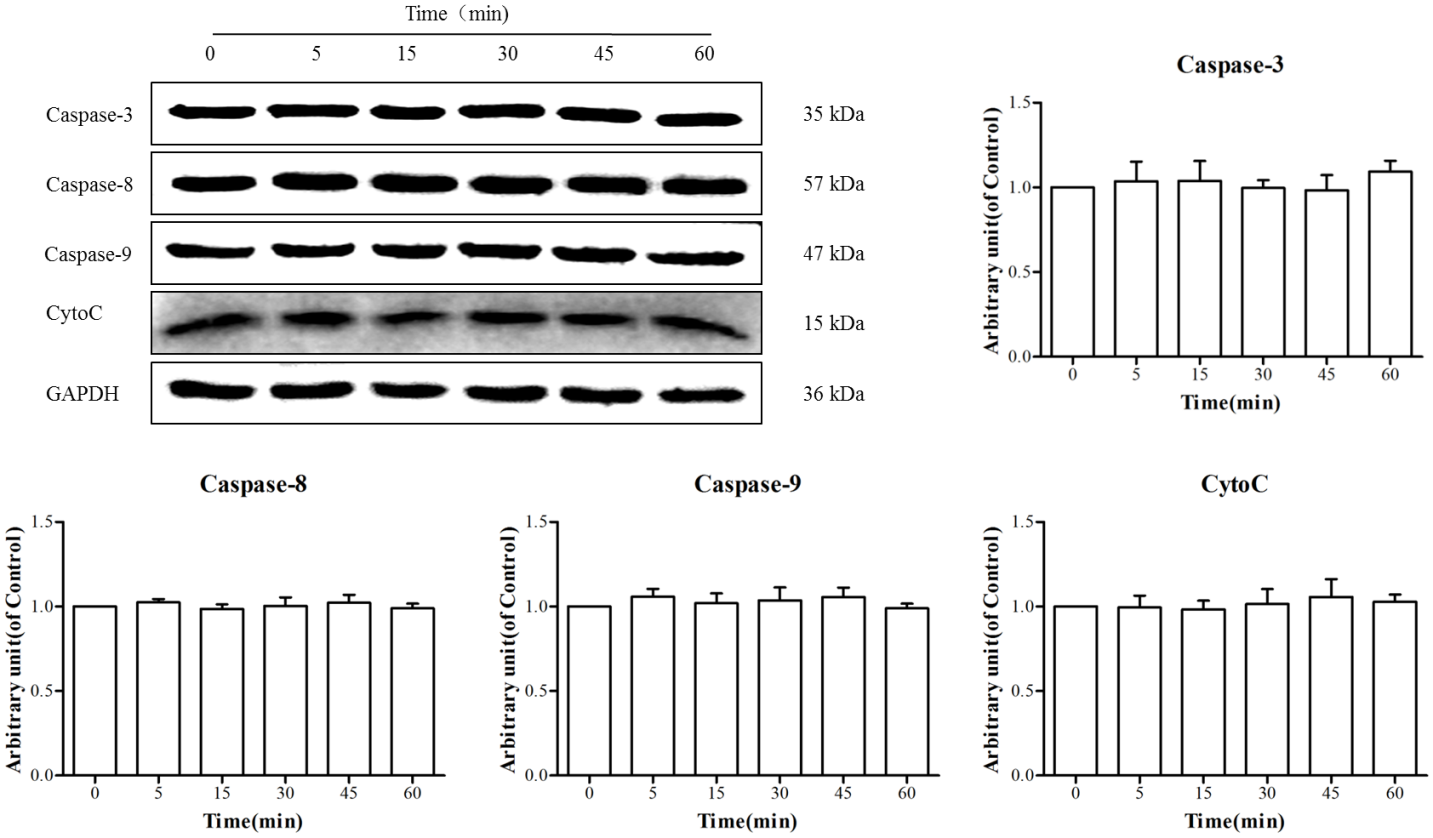
Responses of the downstream signaling cascade (Caspase-3, 8, 9 and CytoC) to TE (1 μg/ml). HUVECs were treated with TE (1 μg/ml) for various durations.

To explore whether TE had an influence on the apoptosis signaling cascade, the effect of TE on apoptosis-related proteins was explored. As shown in Fig 9, after incubation with TE (1 μg/ml), the expression of caspase-3, caspase-8, caspase-9 and CytoC was not affected, which indicated that TE had no effect on the apoptosis signaling pathways.

#### 3.3.2. Effects of TE on the cell signaling pathways in HUVECs by immunofluorescence

To further explore the effects of TE upon the PI3K/Akt, ERK1/2 and JNK MAPK signaling pathways, HUVECs were also analyzed by immunofluorescence staining using corresponding antibodies (green) in the presence or absence of a respective inhibitor. DNA staining with DAPI (blue) was used to define the nuclei.

**Fig 10:**
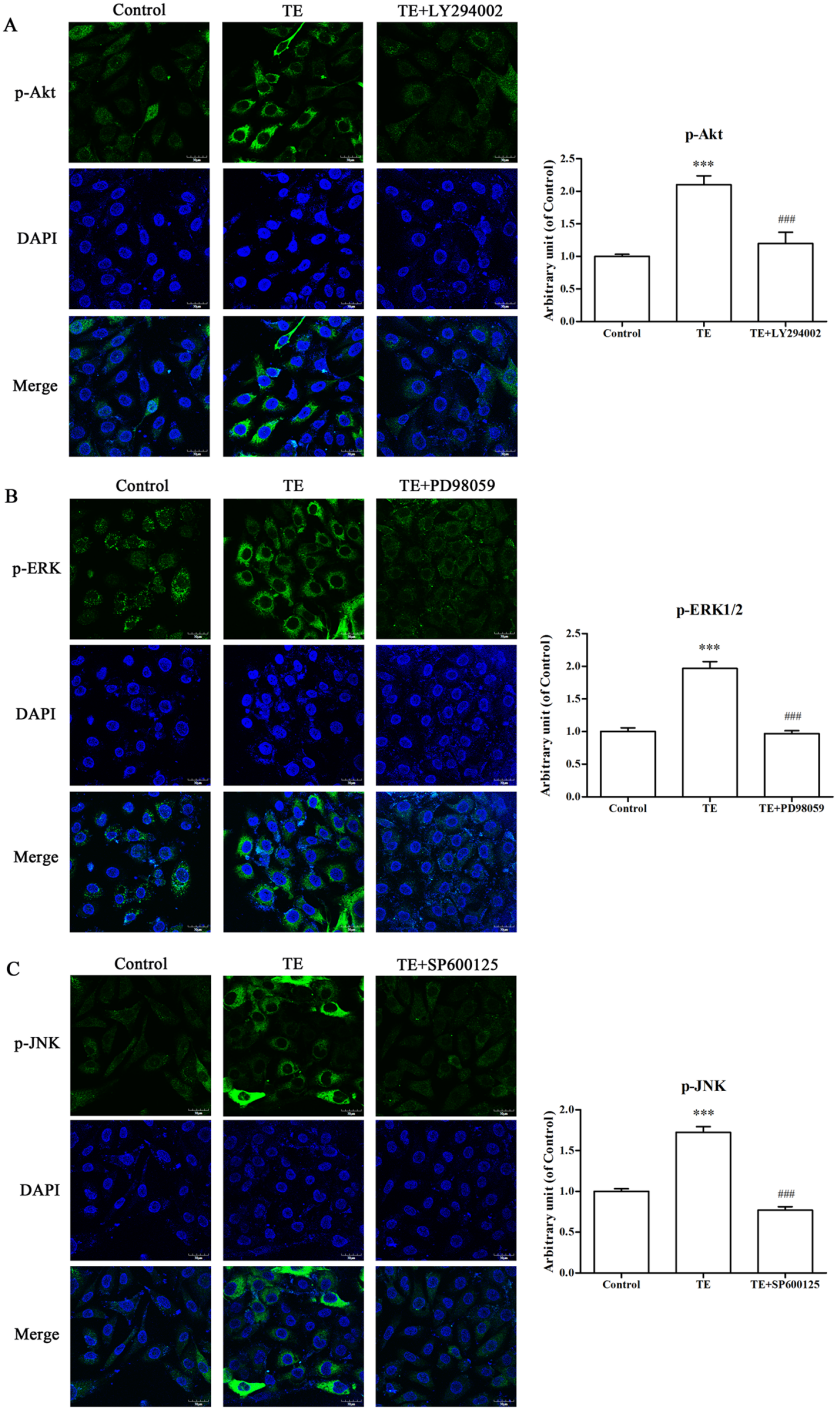
Effects of TE on the phosphorylation of signaling pathways in the presence or absence of inhibitors. A: HUVECs were pretreated with TE (1 μg/ml) for 15 min in the presence or absence of an inhibitor for the PI3K/Akt signaling pathway. B: HUVECs were pretreated with TE (1 μg/ml) for 15 min in the presence or absence of an inhibitor for the ERK1/2 MAPK signaling pathway. C: HUVECs were pretreated with TE (1 μg/ml) for 15 min in the presence or absence of an inhibitor for the JNK MAPK signaling pathway. The images were obtained by a fluorescence microscope at 600 × magnification, scale = 30 μm. ***P < 0.001 *vs*. Control; ^###^*P <* 0.001 *vs*. TE.

As shown in Fig 10, TE could markedly induce the phosphorylation of Akt, whereas the inhibition of PI3K completely blocked TE-induced Akt phosphorylation. Similarly, the phosphorylation of ERK1/2 and JNK was significantly induced by TE, whereas the corresponding signaling pathway inhibitors could effectively block TE-induced ERK1/2 and JNK phosphorylation. These results further confirmed that TE could activate the PI3K/Akt, ERK1/2 and JNK MAPK signaling pathways.

### 3.4. Effects of signaling pathway inhibitors on the expression of TE-induced cell cycle proteins in HUVECs

#### 3.4.1. Effects of signaling pathway inhibitors on the expression of TE-induced cell cycle proteins in HUVECs by western blotting

Since TE could activate the PI3K/Akt, ERK1/2 and JNK MAPK signaling pathways, the corresponding pathway inhibitors were selected to investigate their roles in TE-induced expression of cell cycle proteins.

**Fig 11:**
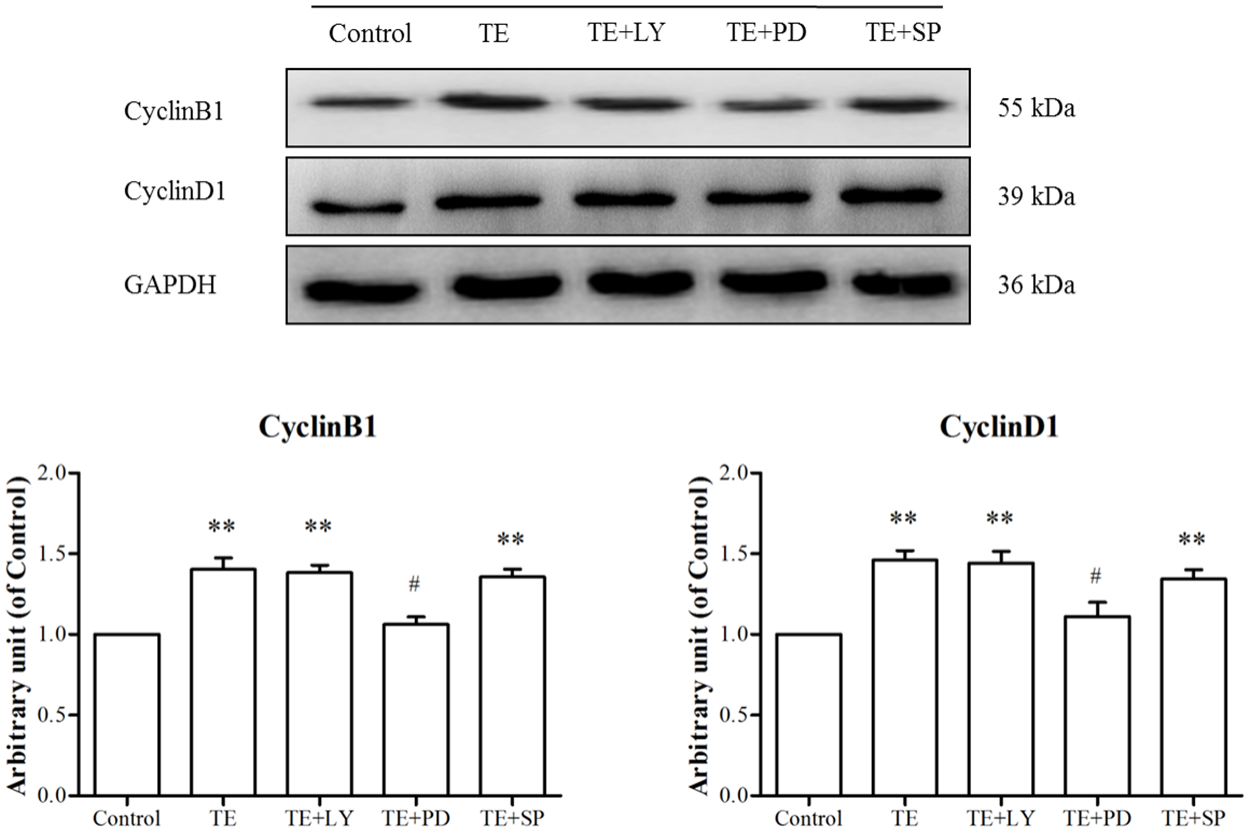
Effects of signaling pathway inhibitors on the TE-induced expression of CyclinB1 and CyclinD1. HUVECs were pretreated with TE (1 μg/ml) for 30 min in the presence or absence of inhibitors of Akt, ERK1/2 and JNK MAPK. LY: LY294002, PD: PD98059, SP: SP600125, Bay: Bay11-7082; **P < 0.01 *vs*. Control; ^#^*P <* 0.05 *vs*. TE.

As shown in Fig 11, the TE-induced expression of CyclinB1 and CyclinD1 was markedly blocked by PD98059, whereas neither LY294002 nor SP600125 could reduce the expression of CyclinB1 and CyclinD1, which demonstrated that the ERK1/2 MAPK signaling pathway was involved in TE-induced cell cycle protein expression in HUVECs.

#### 3.4.2. Effects of signaling pathway inhibitors on the expression of TE-induced cell cycle proteins in HUVECs by immunofluorescence

**Fig 12:**
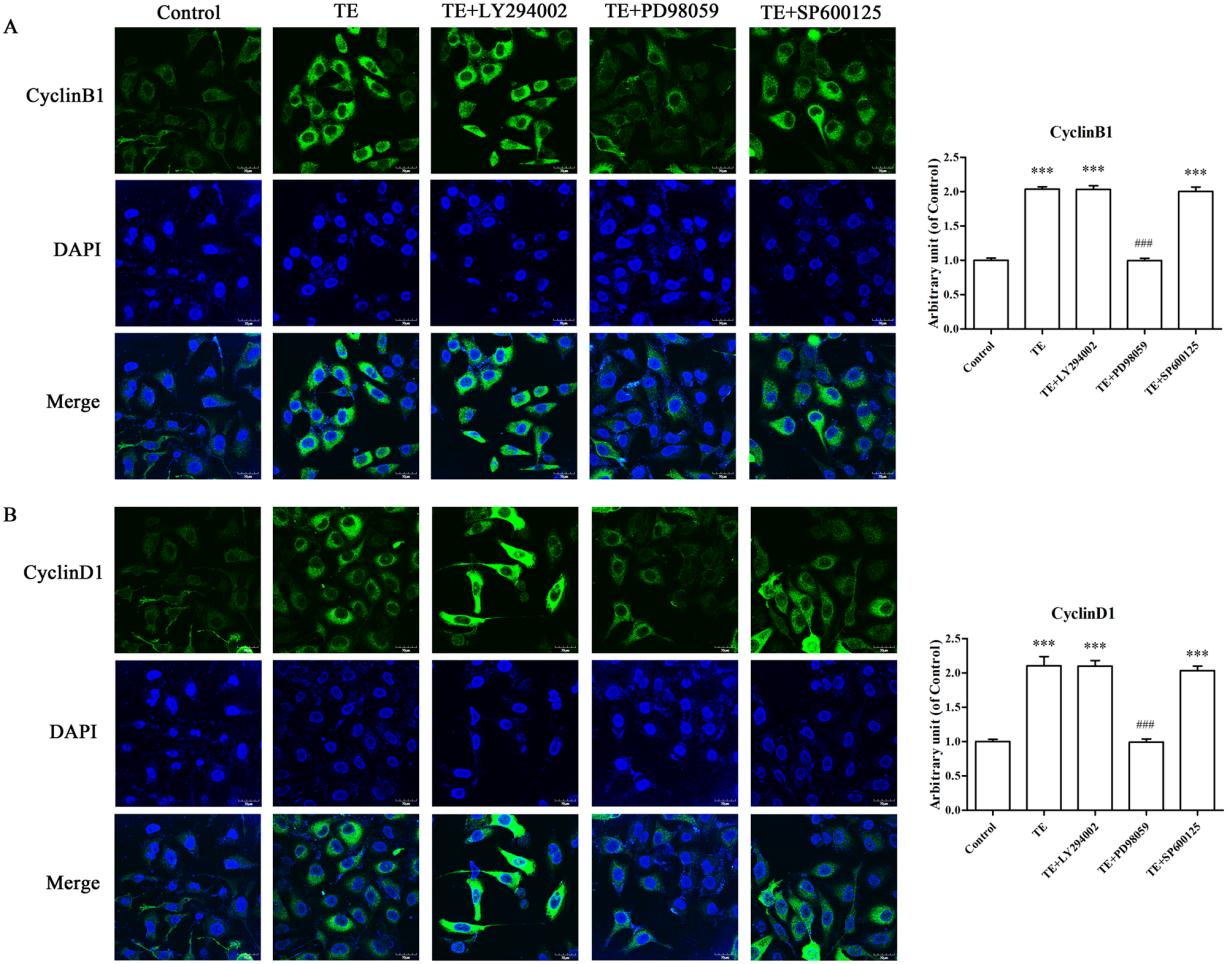
Effects of signaling pathway inhibitors on the TE-induced expression of CyclinB1 and CyclinD1. A: The expression of CyclinB1. B: The expression of CyclinD1. HUVECs were pretreated with TE (1 μg/ml) for 30 min in the presence or absence of inhibitors for Akt, ERK1/2 and JNK MAPK. Images were obtained by a fluorescence microscope at 600 × magnification, scale = 30 μm. ***P < 0.001 *vs*. Control; ^###^*P <* 0.001 *vs*. TE.

As shown in Fig 12, TE dramatically increased the expression of CyclinB1 and CyclinD1. However, the effect was markedly blocked by the pretreatment of PD98059 but not affected by LY294002 or SP600125, which further confirmed that the ERK1/2 MAPK signaling pathway was involved in TE-induced cell cycle protein expression in HUVECs.

### 3.5. Effects of TE on wound healing

Since the ERK1/2 MAPK signaling pathway was involved in TE-induced cell cycle protein expression, we further investigated whether TE affected cell migration in the presence or absence of the ERK1/2 MAPK inhibitor by a wound healing assay.

**Fig 13:**
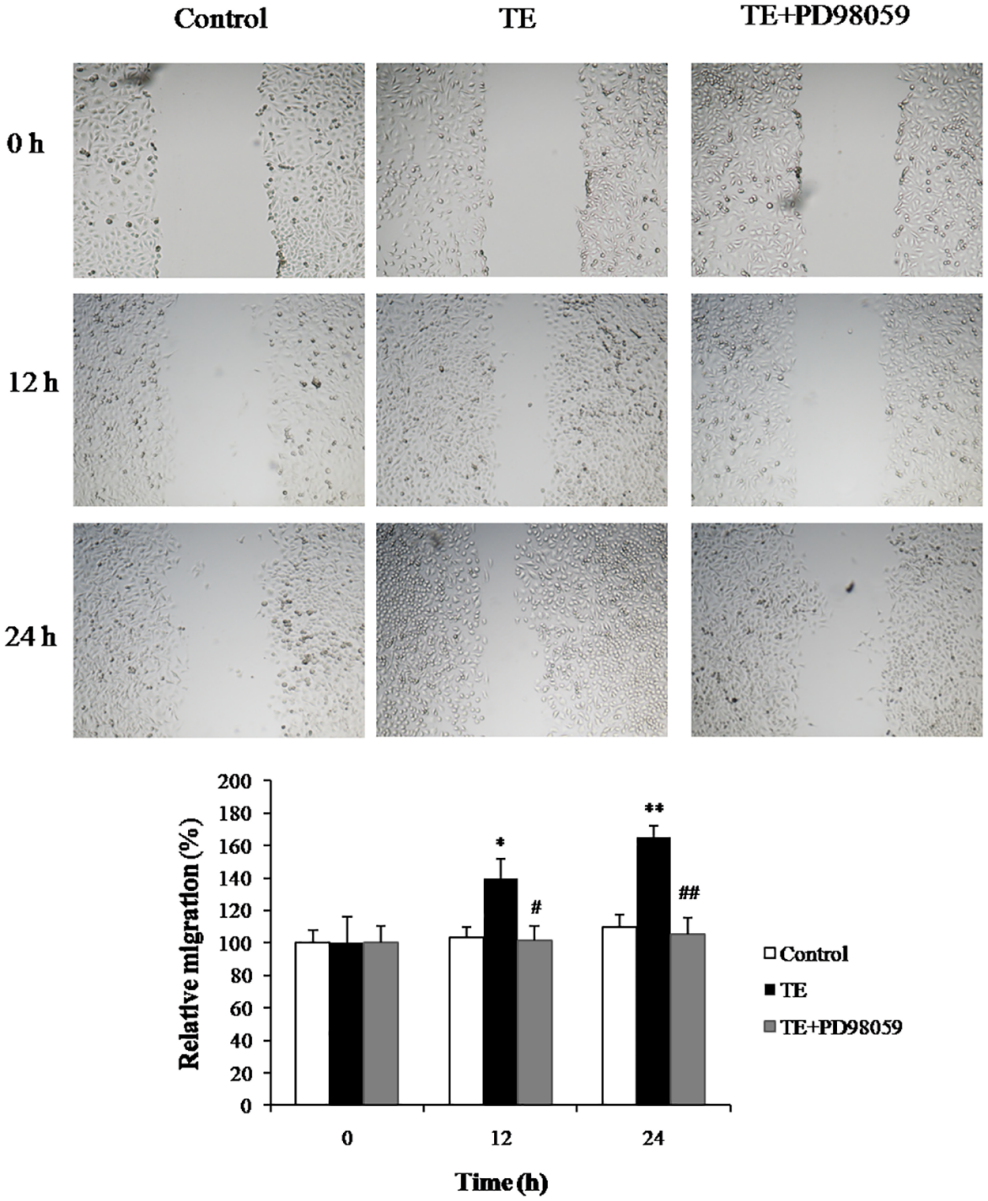
Effects of TE on cell migration in the presence or absence of the ERK1/2 MAPK inhibitor in HUVECs. The images were obtained by a fluorescence microscope at 100 × magnification. *P < 0.05, **P < 0.01 *vs*. Control; ^#^*P <* 0.05, ^##^*P <* 0.01 *vs*. TE.

As shown in Fig 13, TE markedly increased cell migration in a time-dependent manner in HUVECs. The relative migration was approximately 139% at 12 h and 165% at 24 h. However, the TE-enhanced migration ability was markedly attenuated by PD98059, which demonstrated that the ERK1/2 MAPK signaling pathway was involved in TE-promoted endothelial cell migration.

## 4. Discussion

The repair of wounds is one of the most complex biological processes in human life. After an injury, multiple biological pathways are immediately activated and synchronized to respond [15]. There are three classic stages in wound healing, namely, the inflammatory, proliferative, and remodeling phases. The first stage of wound healing includes hemostasis and the activation of inflammatory pathways and the immune system. The intermediate stage involves the recovery of the wound surface, the formation of granulation tissue and the restoration of the vascular network. Late-stage healing involves the remodeling of the extracellular matrix resulting in scar formation and the restoration of the barrier [3, 13, 14]. The ability to respond to injury and to repair tissue is a fundamental property of all multicellular organisms. However, there is tremendous diversity in how this process occurs. Therefore, studying wound repair in various phyla could improve our understanding in humans and might help to identify relevant molecules or pathways that can be targeted to promote wound healing [3].

Jellyfish lack well-developed organs, but they have evolved the molecular and cellular components needed to develop a remarkable body reorganization throughout their lifecycle. A high rate of proliferation has been found in some body parts (especially tentacles) in all life stages including metamorphosing polyps, juvenile medusa and adult medusa, where growth has arrested [21]. However, although jellyfish are easily attacked by other marine organisms in the marine environment, they are also capable of a quick response to various injuries and achieve rapid wound healing without the formation of visible scar tissue [7]. These phenomena imply that jellyfish may contain some bioactive components that can promote cell proliferation and facilitate wound healing.

In our previous study and to get an overview of the novel toxins and bioactive components from jellyfish, we performed *de novo* transcriptome sequencing and identified a great number of transcripts of the tentacle of the jellyfish *C*. *capillata*. Among these transcripts, four sequences exhibited a similarity to the identified VEGFs [22]. The functions of VEGFs in wound repair has been extensively studied including angiogenesis, wound closure, epidermal repair, and granulation tissue formation [17]. These effects are mediated by binding to tyrosine kinase receptors (i.e., the VEGFRs) located on endothelial cells, involving the stimulation of cell proliferation, migration, survival, tube formation and regulation of vascular permeability [17, 23]. Despite the absence of blood vessels or blood cells, VEGF/VEGFR signaling has also been found in invertebrates. The Seipel group [24] reported the presence of VEGF and VEGFR homologous genes in the jellyfish *Podocoryne carnea*, and their results indicated that the highest levels of the VEGF homolog were detected in the muscles and endodermal layer of the tentacles and gastrovascular channels, whereas the highest levels of the VEGFR homolog occurred in the endodermal layer of the tentacles. Therefore, in the present study and using the TE from *C*. *capillata* as the sample, we aimed to investigate the effects and molecular mechanisms of TE on cell proliferation and migration in HUVECs.

First, based on the CCK-8 assay, we demonstrated that TE promoted cell proliferation but not cell death at the concentration of 1 μg/ml for various durations. Moreover, the cell cycle analysis revealed that TE (1 μg/ml) could induce the transition of cells from the G1-phase to the S/G2-phase of the cell cycle and increase the expression of cell cycle proteins (CyclinB1 and CyclinD1), which suggests that TE could induce cell cycle protein expression, speed up cell cycle progression, and ultimately regulate cell proliferation.

It has been known that the PI3K/Akt signaling pathway plays an important regulatory role in the survival, proliferation and migration in many cells [25, 26]. Therefore, we examined the effect of TE on the PI3K/Akt signaling pathway. The results demonstrated that Akt phosphorylation was increased by TE (1 μg/ml), whereas PI3K inhibitor LY294002 completely inhibited the TE-induced phosphorylation of Akt, which suggests that TE could activate the PI3K/Akt signaling pathway. However, MAPK signaling is also considered one of the critical molecular events for the proliferation, survival and migration of vascular endothelial cells in VEGF-induced angiogenesis [27, 28]. Therefore, the phosphorylated status of the MAPK family members (ERK1/2, JNK and p38) was also detected following TE treatment, and the results showed that TE (1 μg/ml) induced the phosphorylated activation of ERK1/2 and JNK but not p38 kinase. Moreover, the inhibitors of ERK1/2 and JNK completely blocked TE-induced phosphorylation, which further confirmed that TE could activate the ERK1/2 and JNK MAPK signaling pathways. Based on these findings, we also detected the phosphorylated status of Akt, ERK1/2 and JNK in HUVECs via immunofluorescence. In agreement with the results by the western blot technique, the immunofluorescence assay indicated that TE-induced phosphorylation of Akt, ERK1/2 and JNK was completely blocked by their respective inhibitors, such as LY294002, PD98059 and SP600125, which further confirmed that TE could activate the PI3K/Akt, ERK1/2 and JNK signaling pathways.

There are several important transcriptional factors involved in cell growth, among which NF-κB is considered closely associated with neovascularization [29]. According to some reports, NF-κB is involved in the tubular morphogenesis of human vascular endothelial cells *in vitro* [30] and is required for retinal angiogenesis *in vivo* [31]. Thus, we also assessed the effects of TE on NF-κB signaling in HUVECs. However, the results indicate that TE (1 μg/ml) treatment seemed to have no effect on the expression of p-NF-κB or p-IκBα in HUVECs, which indicated that TE could not activate the NF-κB signaling pathway. However, it is well-established that caspase activation plays a central role in the execution of apoptosis [32]. Therefore, we also investigated the effects of TE on cell apoptosis signaling pathways, and the results showed that TE (1 μg/ml) treatment did not appear to activate apoptosis-related proteins such as caspase-3, caspase-8, caspase-9 and CytoC. Taken together, these results indicate that multiple signaling pathways were activated by TE in HUVECs following TE treatment.

We performed additional experiments to further establish the role of the PI3K/Akt, ERK1/2 and JNK signaling pathways in TE-induced proliferative effects in HUVECs. Our results showed that the TE-induced expression of cell cycle proteins (CyclinB1 and CyclinD1) was markedly blocked by ERK1/2 inhibitor PD98059 but not by PI3K/Akt or JNK inhibitors, and similar results were also observed in the immunofluorescence assay. Therefore, we propose that only the ERK1/2 and MAPK signaling pathways were involved in the regulation of TE-induced proliferation in HUVECs. However, it has been reported that ERK1/2 is involved in regulating cell migration; the duration and magnitude of ERK1/2 activation associates with cell motility [26]; and the inhibition of ERK1/2 activation results in markedly reduced movement of epithelial and endothelial cells in wound healing experiments [33, 34]. Therefore, in this study, we also performed a wound healing assay and found that the TE-enhanced migration ability of HUVECs was notably impaired when the ERK1/2 activity was inhibited by PD98059, which implied that the ERK1/2 signal transduction was involved in the regulation of TE-promoted endothelial cell migration. However, the detailed signal transduction cascade underlying TE-induced cell migration remains to be elucidated.

## 5. Conclusion

In this study, we provided experimental evidence that TE from *C*. *capillata* could significantly promote the proliferation and migration in HUVECs, which may contribute to the healing-promoting effect of TE. We found that TE may induce cell cycle protein expression, speed up cell cycle progression, and ultimately regulate cell proliferation. In addition, we demonstrated that TE could activate the PI3K/Akt, ERK1/2 and JNK signaling pathways, and the ERK1/2 signal transduction among them was involved in the regulation of TE-induced proliferation and migration in HUVECs. These findings indicate the desired prospects of TE in regulating wound repair and set the groundwork for further isolation and purification of bioactive components (i.e., the VEGFs) from the jellyfish, which may be hopeful to improve human wound repair in unfavorable conditions.
